# Study on Low-Temperature Cracking Performance of Asphalt under Heat and Light Together Conditions

**DOI:** 10.3390/ma13071541

**Published:** 2020-03-27

**Authors:** Limin Li, Zhaoyang Guo, Longfei Ran, Jiewen Zhang

**Affiliations:** 1 School of Civil and Environmental Engineering, Hunan University of Science and Engineering, Yongzhou 425199, China; 2 Inner Mongolia Communications Construction Engineering Quality Supervision Bureau, Hohhot 010050, China; 3 School of Civil Engineering, Chongqing Jiaotong University, Chongqing 400074, China

**Keywords:** heat and light together, asphalt, low-temperature cracking performance, aging

## Abstract

The low-temperature cracking performance of asphalt is considered one of the main deteriorations in asphalt pavements. However, there have been few studies on the low-temperature cracking performance of asphalt under heat and light together. Hence, the ductility test, bending beam rheometer (BBR) test, and asphalt composition analysis test are combined to investigate the low-temperature cracking performance under heat and light together based on the climatic conditions of China. The styrene–butadiene–styrene block copolymer (SBS)-modified asphalt binders were prepared with different modifier types and base asphalt in this research. The results show that the low-temperature cracking resistance of asphalt reduces under heat and light together. It is obviously reduced at the early stage, and it becomes worse with the increase of the aging time, temperature, and ultraviolet (UV) intensity. The asphalt composition has a significant impact on its low-temperature cracking performance, and the SBS modifier can improve the low-temperature cracking resistance of asphalt. The rational selection of base asphalt and modifier can improve the low-temperature cracking performance of asphalt. Under heat and light together, whether base asphalt or modified asphalt, the change trends of their ductility and component content are similar. Therefore, to improve the anti-cracking ability of the asphalt pavement, it is suggested to use the ductility of asphalt aged by heat and light together for 15 days as the evaluation index of the low-temperature cracking performance of asphalt, and asphalt should be selected according to the temperature and UV intensity of the asphalt pavement use area.

## 1. Introduction

### 1.1. Background

Asphalt pavement cracking will cause the surface water to run into the pavement, which will cause many issues, such as loose, potholes, and stripping. Water into the pavement will also soften the base course and the subgrade, which will cause causes different issues, such as steps, slurry pumping, and mesh cracking [1]. Cracking will accelerate the asphalt pavement damage as well as decreasing its service life [2,3]. To solve the cracking failure of asphalt pavement, the researchers conducted much work on the cracking of asphalt pavement and asphalt mixture. Mao [4] studied the crack initiation mechanism and propagation behavior of asphalt pavements, and the results showed that the mechanism for crack propagation was primarily tensile. Yan [5] investigated the cracking mechanism and prevention measures of top–down cracks on a flexible base asphalt pavement in a seasonal frozen area and found that the traffic load, environmental factors, and asphalt aging were the major reasons for top–down cracking. Research on the causes, effects, and cures of top–down cracking in asphalt pavement has been conducted by Donna et al. [6], and the results showed that the mixture with higher asphalt content would not be as prone to segregate. Wang [7] analyzed the crack problem of the asphalt pavement under UV light aging and claimed that asphalt pavement was more prone to cracks because of the effect of UV. Ramos et al. [8] reviewed the methods to measure the fatigue damage resistance of asphalt binders and put forward their suggested method. Wu et al. [9] evaluated the aging effect of UV radiation on the asphalt mixture and found that UV radiation significantly affected the asphalt semi-circular bending strength of asphalt concrete, it decreased the −10 °C bending strength of asphalt concrete, and the longer the exposure duration was, the more obvious the aging effect. Behnia et al. [10] reviewed the approach to evaluating the low-temperature cracking of asphalt and found that acoustic emission could assess the low-temperature cracking performance of asphalt. Although lots of investigations have been performed for the cracking of asphalt pavement and asphalt mixture, research rarely focuses on the effect of heat and light together.

On the other hand, lots of investigations have been conducted for the low-temperature performance of asphalt materials. The low-temperature performance and evaluation method of asphalt were implemented by several researchers [11,12,13,14,15], although they didn’t consider the influence of light and heat together. Zhou et al. [16] studied styrene–butadiene–styrene (SBS)-modified asphalts’ anti-cracking characteristics by using the rolling thin film oven (RTFO) and the Pressure Aging Vessel (PAV) residue based on Strategic Highway Research Program (SHRP) methods. It was shown that correlation was very good among the complex shear modulus, phase angle, fatigue cracking factor, creep stiffness, m value, and temperature in the range of wider temperature. The cracking characteristics of three modified and nine unmodified asphalt binders aged by different testing methodologies i.e., an UV route and a thermal route with changing exposition time by Patrícia et al. [17], and it was found that the critical block-cracking zone could be reached by the two routes, being the oxidative effect that happens in the field more evident in the UV radiation route. The carbonyl index was very sensitive to UV aging and presented in general larger values than the other aging strategies. Zhang et al. investigated the effect of long-term laboratory aging on the rheological properties and cracking resistance of polymer-modified asphalt binders at intermediate and low-temperature ranges and analyzed different evaluation indexes [18]. Glotova et al. [19] researched the effect of UV aging on an asphalt binder by testing the composition contents, structures, and the chemical properties of asphalt binder before and after aging, and the results showed that the type of light irradiation had a close relationship with the photo-oxidation aging speed, and UV radiation had obvious degeneration effects on these properties. The aging effect of three types of aging methods—namely UV radiation, pressure aging vessel (PAV), and thin film oven test (TFOT) was investigated by Zhang et al. [20], and they found that PAV aging and UV aging have more obvious effects on the degradation of styrene–butadiene–styrene (SBS) polymer. The results showed that the studied binders tended to be more viscous at relatively low loading frequencies and harder after aging, and the ability to relax internal stress declined. The UV light plays a great role in the photo-oxidation of bitumen and makes the surface layer of asphalt pavement stiffer and brittle [21,22,23]. However, the effect on the cracking resistance of asphalt heat and light together has been rarely performed to date.

In fact, the low-temperature cracking resistance of asphalt has a vital influence on the anti-cracking performance of asphalt pavement [24,25,26]. Meanwhile, due to asphalt’s temperature sensitivity, light and heat affect the crack resistance of asphalt during the asphalt pavement construction and service life [27]. The PAV test and the RTFOT (Rotary thin film oven test) are commonly used in the world to simulate the long-term aging behavior and short-term thermo-oxidative aging of bitumen [21,28]. However, due to ignoring the effect of UV radiation, the correlation between actual pavement aging and PAV simulation aging is not significant. At present, understanding of the cracking performance of asphalt under heat and light together is very limited. Hence, to solve the problem of asphalt pavement cracking, it is necessary to investigate systematically the low-temperature cracking performance of asphalt under heat and light together based on the environmental conditions of asphalt pavement.

### 1.2. Objectives

The main objective of the paper is to assess the effect of heat and light together on the low-temperature behavior of asphalt mixtures expressed under the climatic conditions of China in terms of the ductility curves (ductility test), the parameters of the bending beam rheometer (BBR) test, and the compositions (asphalt composition analysis test) of the asphalt aged under heat and light together. 

## 2. Materials

The SBS modifiers used in the research are the 501s branched and the 4402 linear modifier produced by Dongguan Yuetai New Material Co., Ltd., Guangdong, China, and their characteristic properties are illustrated in Table 1. Zhonghai AH-70 (ZH-70) asphalt and South Korea AH-70 (SK-70) asphalt were employed as the base asphalt sample, and their characteristic properties are listed in Table 2. Based on the existing research results [29,30] and economy, the dosage of SBS modifiers of modified asphalt was 5%. The modified asphalts were prepared using an FM300 High Shear Dispersing Emulsifier (FLUKO, Shanghai, China). Prior to shearing with the dispersing emulsifier, the base asphalt was preheated to 170–180 °C in a small container. Then, according to the required dose, the 501s branched or 4402 linear modifier was gradually added to the base asphalt. Meanwhile, the mix was heated and maintained at 175–180 °C, and it was melted and mixed for 30 minutes. Afterward, the mix was stirred with a high-shear mixer for 40–60 min with a shearing rate of 3000–5000 r/min at 175–180 °C. At last, the mix was put into the oven for 2 h at 175 °C. The characteristic properties of four kinds of modified asphalt for the test are given in Table 3.

**Figure 1:**
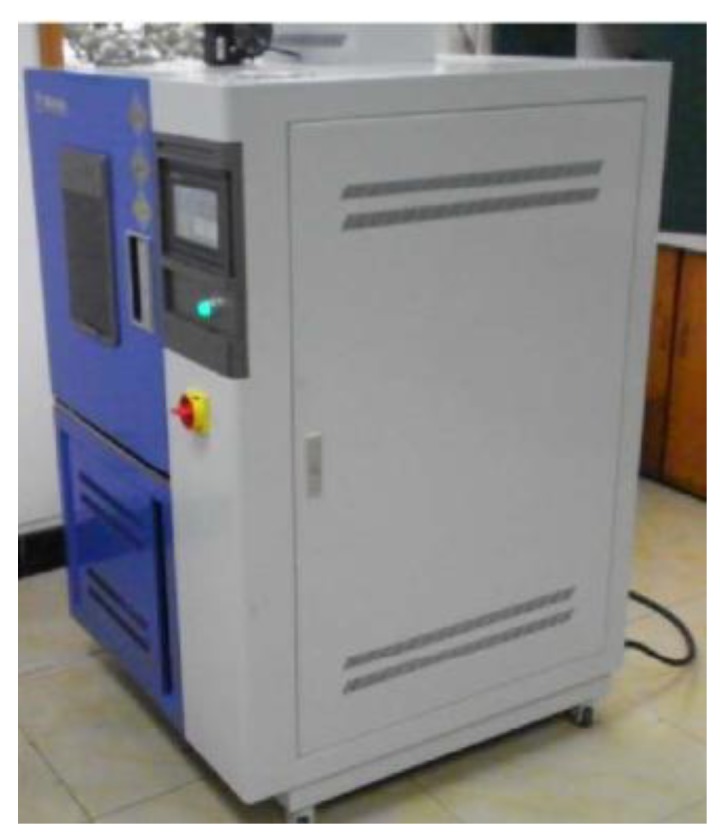
RG-80 asphalt aging test chamber.

**Figure 2:**
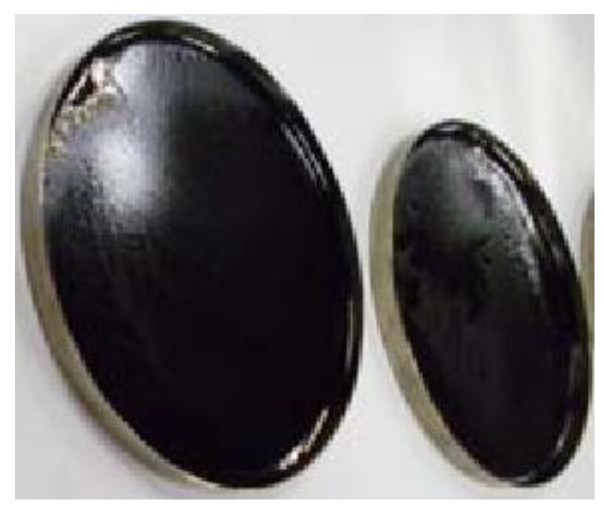
Asphalt sample.

The aging confronting asphalt during asphalt construction was simulated by using short-term aging addressed by RTFO (85 min, 163 °C and air flow at 4 L/min) according to ASTM D2872 [31]. The long-term aging confronting asphalt during the use of asphalt pavement was simulated by using PAV aging (air pressure at 2.1 MPa). The long-term aging of asphalt under heat and light together was done using a RG-80 asphalt aging test chamber manufactured independently, as shown in Figure 1. Considering the maximum ground temperature of 65 to 75 °C in most areas of China and the strong heat absorption ability of asphalt pavement, the maximum temperature of the aging test was set to 70 °C [32]. Considering the aging of asphalt pavement of sunlight caused by the wavelength range of 260–410 nm UV radiation [23,33], the high-pressure mercury lamp with a radiation spectrum ranging from 250 to 440 nm was used as the light source of the RG-80 asphalt aging test chamber. The UV intensity was controlled by the power, quantity, and voltage of the high-pressure mercury lamp, and it was measured by the UV365 UV radiometer (Beijing Normal University Photoelectric instrument Company, Beijing, China). Based on the previous study, the results by Zhang and Ran [32,33], the maximum UV intensity of 40 W/m^2^ and the longest time of 15 days were adopted for the aging test. To meet the requirement of UV light aging, the melted asphalt for the aging test was put into a flat bottom disk with an inner diameter of 140 mm and a height of 9.5 mm, as shown in Figure 2, and the asphalt weight of the flat bottom disk was 30 g.

## 3. Low-Temperature Cracking Performance of Asphalt

### 3.1. Ductility Test

**Figure 3:**
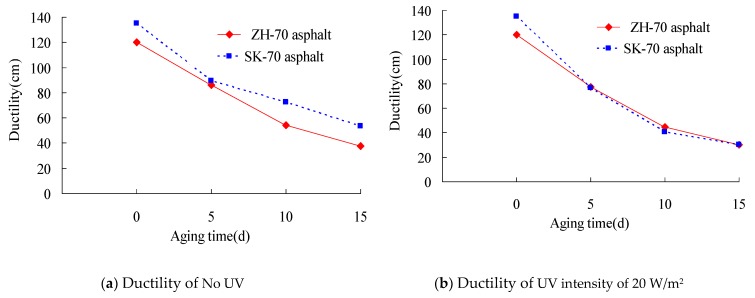

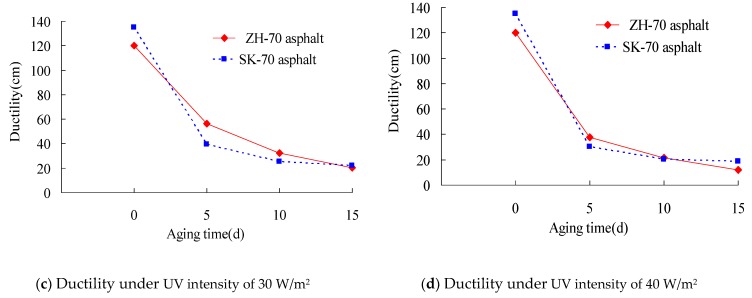
Relationship between ductility and aging time.

**Figure 4:**
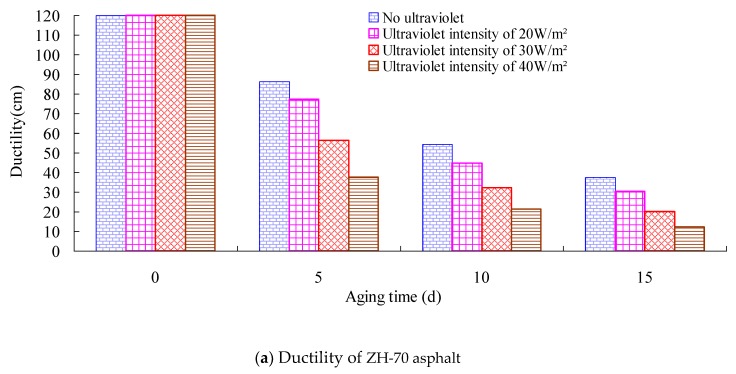

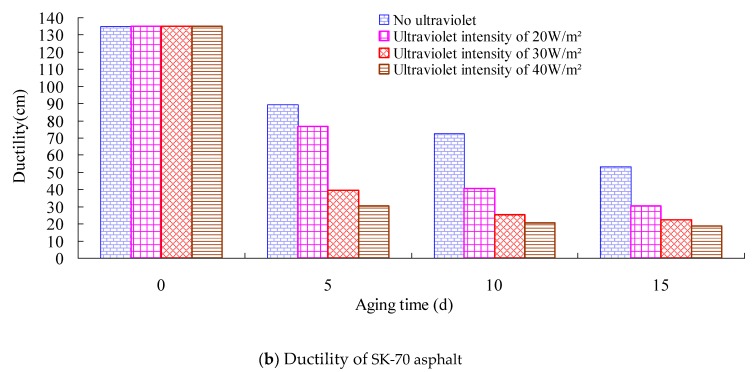
Relationship between ductility and aging time for different UV intensity.

Ductility can affect the flexibility of asphalt, and it can evaluate the tensile deformation ability of the bituminous binder. The smaller the ductility value of asphalt is, the better its crack resistance. Firstly, the index tests of ZH-70 and SK-70 asphalt for the 15 °C ductility under different aging conditions were conducted in the research. The long-term aging of asphalt under heat and light together was conducted, and its aging temperature was set to 70 °C. Its aging time was 0 day, 5 days, 10 days, and 15 days, respectively, and its aging UV intensity was 0 W/m^2^, 20 W/m^2^, 30 W/m^2^, and 40 W/m^2^, respectively. The test results are shown in Figure 3 and Figure 4. The ductility test was carried out according to the standard testing methods of bitumen and bituminous mixtures for highway engineering (JTG E20–2011) in China.

It can be seen from Figure 3 and Figure 4 that within the 5-day aging time, the ductility value of asphalt decreased at a rapid speed, and as the extension of aging time, the reduction rate of the ductility value gradually slowed down. Compared with the ductility value of the 0-day aging time, under the aging UV intensity of 40 W/m^2^, the ductility values of ZH-70 and SK-70 asphalt aged for 5 days were decreased respectively by 68.6% and 77.6%. Furthermore, under the same conditions, the stronger the aging UV intensity, the bigger the ductility value of asphalt decreased. Under the aging UV intensity of 0 W/m^2^, compared with the ductility value of the 0-day aging time, the ductility values of ZH-70 asphalt and SK-70 asphalt aged for 15 days were decreased by 68.8% and 60.4% respectively, and under the aging UV intensity of 40 W/m^2^, they were decreased by 89.8% and 86.2%, respectively. 

**Figure 5:**
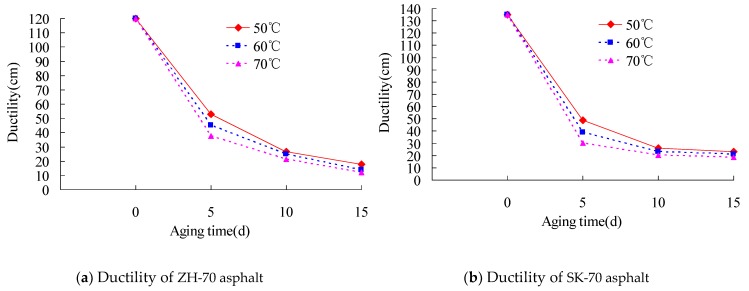
Relationship between ductility and aging times for different temperatures.

Then, the index tests of ZH-70 and SK-70 asphalt for 15 °C ductility under different aging temperatures were conducted. The long-term aging of asphalt under heat and light together was performed, and its aging UV intensity was set to 40 W/m^2^. Its aging time was set to 0 day, 5 days, 10 days, and 15 days, respectively, and its aging temperature was set to 50 °C, 60 °C, and 70 °C, respectively. The results are presented in Figure 5.

As observed in Figure 5, the ductility value of asphalt decreased with the increase of aging time. However, its decrease rate slowed down. Meanwhile, the ductility value of asphalt was also clearly decreased within the aging time of 5 days. At the aging temperature of 60 °C, compared with the ductility value of the aging time of 0 day, the ductility values of ZH-70 and SK-70 asphalt aged for 5 days were decreased respectively by 62.5% and 71.1%. Furthermore, under the same conditions, the higher the aging temperature, the bigger the ductility value of the asphalt that decreased. At the aging temperature of 50 °C, compared with the ductility value of the aging time of 0 day, the ductility values of ZH-70 and SK-70 asphalt for the aging time of 15 days were decreased by respectively 85% and 82.9%, and at the aging temperature of 60 °C, they were decreased by 88.3% and 84.4 %, respectively. 

**Figure 6:**
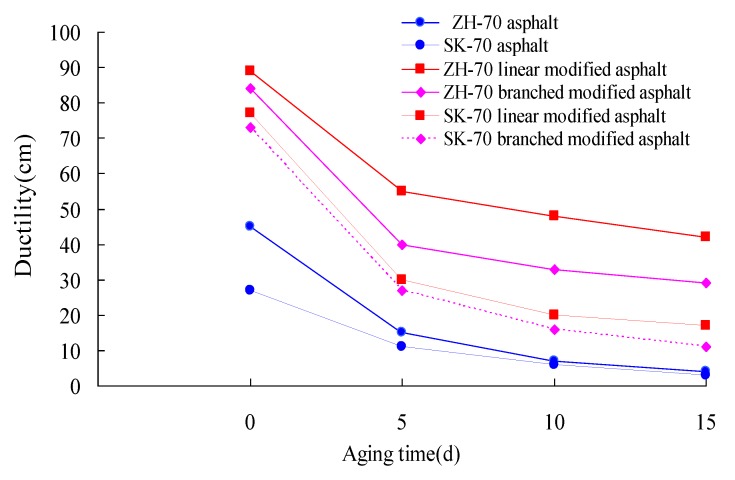
Ductility and aging times for different asphalts.

**Figure 7:**
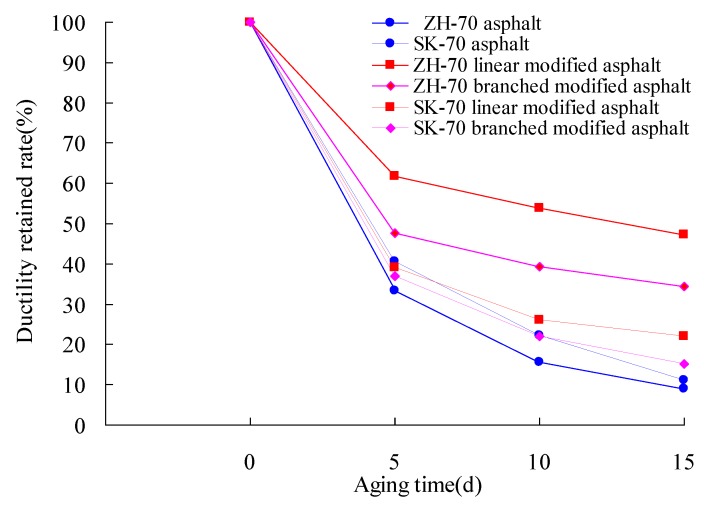
Ductility retained rates and aging times for different asphalts.

Lastly, under the aging UV intensity of 40 W/m^2^, the index tests of six kinds of asphalt for the 10 °C ductility were conducted at the aging temperature of 70 °C. The long-term aging of asphalt under a heat and light coupling condition was performed, and its aging time was 0 day, 5 days, 10 days, and 15 days, respectively. The ductility retained rates of asphalt under different aging times were calculated. The test results are given in Figure 6 and Figure 7.

It can be seen from Figure 6 and Figure 7 that whether base asphalt or modified asphalt, the change trends of the ductility of asphalt were similar. Their ductility values were decreased at a rapid speed within the aging time of 5 days, and as the extension of aging time, they were gradually decreased. For different types of asphalt, the ductility retained rate was different, and compared with the base asphalt, the ductility value of the modified asphalt was significantly higher than that of the base asphalt. For ZH-70 asphalt, ZH-70 linear-modified asphalt, ZH-70 branched-modified asphalt, SK-70 asphalt, SK-70 linear-modified asphalt, and SK-70 branched-modified asphalt, their ductility values of the aging time of 15 days were respectively 4 cm, 42 cm, 29 cm, 3 cm, 17 cm, and 11 cm, and their ductility retained rates of the 15-day aging time were 9%, 47.2%, 34.5%, 11.1%, 22.1%, and 15.1% respectively. For the same modifier, the ductility value of modified asphalt for different base asphalts was different, too. At the 15-day aging time, for ZH-70 asphalt, the ductility value of its linear-modified asphalt and branched-modified asphalt was 10.5-fold and 7.25-fold higher than that of its base asphalt, respectively, and for SK-70 asphalt, the ductility value of its linear-modified asphalt and branched-modified asphalt was 5.66-fold and 3.66-fold higher than that of its base asphalt respectively. Moreover, for the same modifier, the ductility value of different bases was also different. For the 501s branched modifier and the 4402 linear modifier, their ductility values of ZH-70-modified asphalt aged for 15 days were 2.47-fold and 2.64-fold higher than those of SK-70-modified asphalt, respectively. The different chemical composition of the base asphalt was the possible reason causing the difference.

The ductility test results indicate that heat and light have a great effect on the low-temperature cracking performance of asphalt, and they can obviously reduce the low-temperature cracking performance of asphalt at the early stage. Moreover, the higher the values of the temperature and UV intensity are, the more the low-temperature cracking performance of asphalt is reduced. The asphalt type has an important effect on the low-temperature cracking performance of asphalt under heat and light together, and the rational selection of a base asphalt and modifier can improve the low-temperature cracking performance of asphalt.

### 3.2. Bending Beam Rheometer (BBR) Test

**Figure 8:**
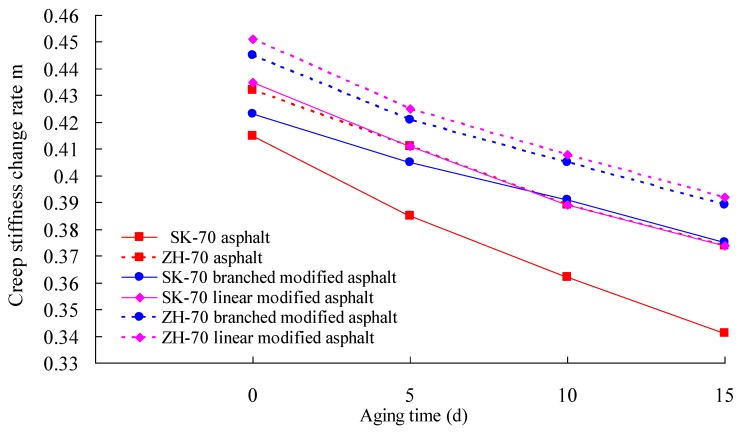
Creep stiffness change rate and aging time.

**Figure 9:**
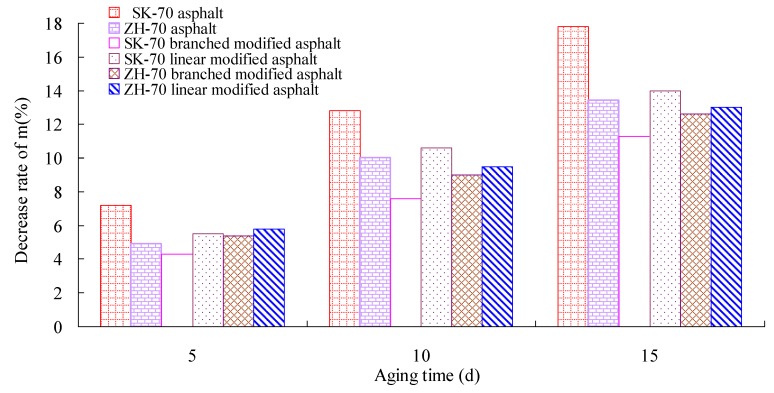
Decrease rate of creep stiffness change rate and aging time.

**Figure 10:**
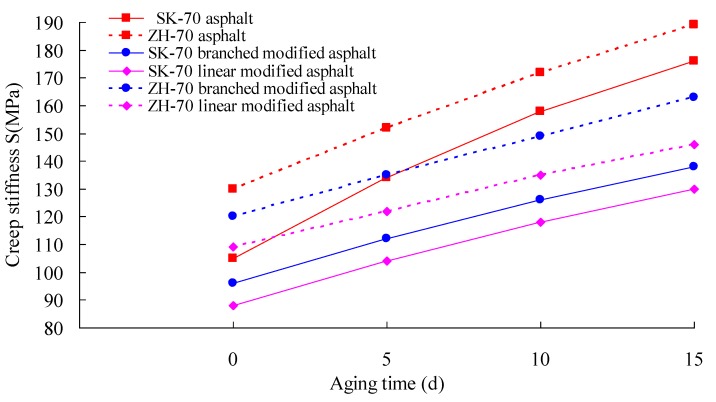
Stiffness modulus and aging time.

**Figure 11:**
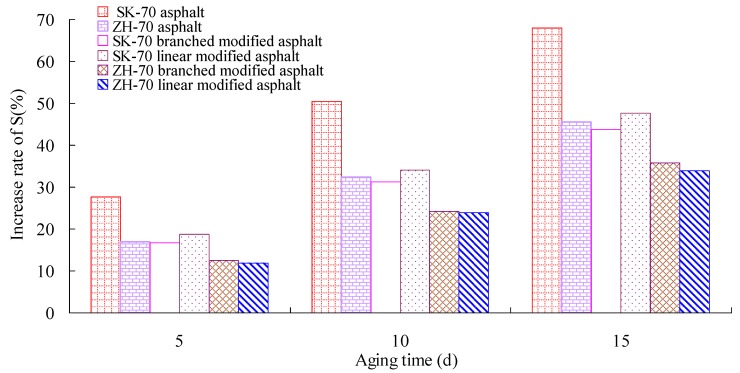
Increase rate of stiffness modulus and aging time.

Firstly, the RTFOT aging test of the asphalt was performed. Then, under a UV intensity of 40 W/m^2^, the long-term aging test of the asphalt aged by RTFOT was conducted at 70° C. Afterward, the aged asphalt was used in the BBR test. According to Superpave specifications, BBR tests of six kinds of asphalt were performed using a BBR 9728-V30 from the Cannon Company, at −12 °C. The creep stiffness change rate m, at 60 s, and the creep stiffness S, are the most important parameters of the BBR test, and they can evaluate the low-temperature crack resistance of asphalt binder. A smaller S-value and a bigger m-value correspond to a better deformation ability and stress relaxation ability, respectively. That is, a smaller S-value and a bigger m-value correspond to a better low-temperature crack resistance of asphalt. The change rates of the S-value and the m-value of asphalt under different aging times were calculated. The test results are given in Figure 8, Figure 9, Figure 10 and Figure 11.

As observed in Figure 8 through Figure 11, whether base asphalt or modified asphalt, the S-value was clearly elevated, and the m-value was obviously reduced with the increase of aging time, indicating that under heat and light together, the obvious embrittlement occurred in asphalt at low temperature, and the low-temperature crack resistance of asphalt was reduced. Moreover, under the same conditions, the m-value of the modified asphalt was bigger than that of the base asphalt, and the S-value of the modified asphalt was smaller than that of the base asphalt. That is, in comparison with the base asphalt, the low-temperature crack performance of modified asphalt was better. For different base asphalts and modifiers, the increase rate of the S-value and the decrease rate of the m-value were also different. Therefore, for the low-temperature crack performance, the heat and light aging have an obvious effect on the low-temperature cracking performance of asphalt; the effect on modified asphalt was smaller than that on base asphalt, and the rational selection of base asphalt and modifier can improve the low-temperature cracking resistance of asphalt.

### 3.3. Asphalt Composition Analysis Test

**Figure 12:**
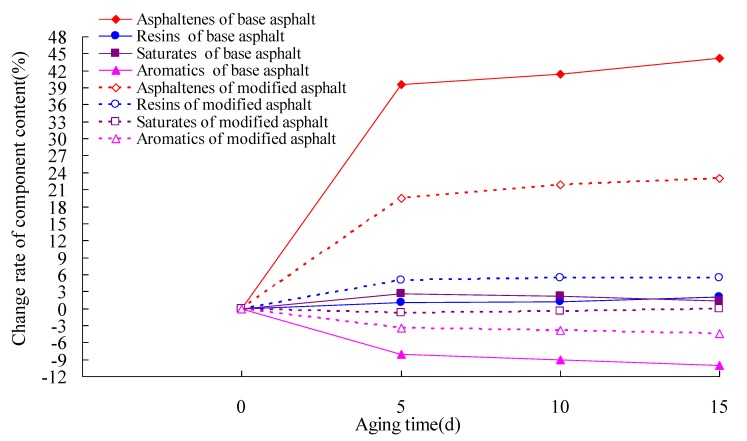
Change rate of component content and aging time for ZH-70 base and modified asphalt.

The asphalt composition has a direct effect on its low-temperature cracking performance [34]. To research the effect of heat and light together on the low-temperature cracking performance of asphalt, four constituent tests of ZH-70 asphalt and ZH-70 linear-modified asphalt were performed, according to the test method for the separation of the asphalt into four fractions (T0618-1993), based on the standard test methods for bitumen and bituminous mixtures for highway engineering (JTG E20-2011) in China [35]. Firstly, the RTFOT aging test of asphalt was conducted. Then, under the aging UV intensity of 40 W/m^2^, the long-term aging test of asphalt aged by RTFOT was performed at 70° C. Afterward, the aged asphalt was used in the asphalt composition analysis test. The change rate of component content (CRC) of asphalt was used for evaluating the content change of the component of asphalt before and after aging, and it can be calculated using the following Equation:

(1) $$CRC=\frac{CCA-CCB}{CCB}\times 100\%$$

where CCA is the content of the component of asphalt after aging, and CCB is the content of asphalt component before aging. The content change rates of the component of asphalt under different aging times were calculated. The results are given in Table 4 and Table 5 and Figure 12.

Table 4 and Table 5 show that in comparison with the base asphalt, before long-term aging, the content of asphaltenes of modified asphalt increased, and its contents of resins, aromatics, and saturates decreased in various degrees, indicating that the SBS modifier can increase the viscosity and surface area of asphalt, because of the high speed shear and swelling, and it can adsorb some colloids and dispersing media. Moreover, the SBS modifier is of a polyphase network structure; after it was added to the base asphalt, a good three-dimensional network structure formed, and the polystyrene chain dispersed in the matrix asphalt phase. Those can improve the elasticity and flexibility of its modified asphalt at low temperature, which may be the reason that the low-temperature cracking resistance of modifier asphalt is better than that of base asphalt. 

It can be seen from Table 4 and Table 5 and Figure 12 that the content of asphaltenes increased significantly, while the content of aromatics clearly decreased with the increase of aging time. Meanwhile, the contents of saturates and resins sometimes increased and sometimes decreased, and the changes of their content were not obvious. Therefore, it can be the main reason that the transformations from aromatics to resins and then from resins to asphaltenes occurred in the aging process of asphalt. The content of resins was affected by the rates of the transformations through aromatics to resins and through resins to asphaltenes. Furthermore, the change rates of asphaltenes and aromatics of modified asphalt were smaller than those of base asphalt, indicating that after the modifier is added to the base asphalt, it can decrease the rate of the transformations through aromatics to resins and then through resins to asphaltenes. As seen in Figure 12, the change of asphaltenes content was the most obvious, and within the 5-day aging time, the four components content varied obviously: as the aging time extended, their change rates slowed down. It can be one of the main reasons that with the increase of aging time, the chain rupturing and ring-opening reactions of asphaltenes will occur and asphaltenes will transform to saturates and aromatics again. 

Under heat and light together, the active bond of asphalt was activated, a polar group was produced by the oxidation reaction, there was intermolecular force enhancing due to the aggregation of the polar groups, macromolecular substances were produced; then, the content of asphaltenes of asphalt increased, which caused the decrease of ductility of the asphalt at low temperature. Moreover, the degradation of the polystyrene chain will cause the decrease of the ductility of modified asphalt at low temperature. Those may be the reason that the low-temperature cracking resistance of asphalt decreased with the increase of aging time under heat and light together.

## 4. Conclusions

In this research, six kinds of asphalt were exposed under heat and light together for 5, 7, and 15 days to investigate the low-temperature cracking performance, and the following conclusions can be obtained.

(1) Under heat and light together, the ductility value of asphalt decreased with the increase of aging time, temperature, and ultraviolet intensity, and it became more obvious within 5 days; the ductility value of modified asphalt was significantly higher than that of base asphalt, and for the same modifier, the ductility value of modified asphalt for different base asphalts was also different, indicating that the asphalt cracking resistance was obviously reduced at the early stage. Moreover, it becomes worse with the increase of the aging time, temperature, and ultraviolet intensity, and the rational selection of base asphalt and modifier could improve the low-temperature cracking performance of asphalt.
(2) Under heat and light together, the creep stiffness S value of asphalt was significantly increased, while the creep stiffness change rate m value of asphalt was obviously reduced with the increase of aging time. Meanwhile, the change rates of the S value and m value of modified asphalt were smaller than those of base asphalt. Hence, heat and light together aging could significantly weaken the low-temperature cracking resistance of asphalt, and they had less effect on the low-temperature cracking resistance of modified asphalt.
(3) Under heat and light together, the content of asphaltenes increased significantly, while the content of aromatics clearly decreased with the increase of aging time. The change rates of asphaltenes and aromatics of modified asphalt were lower than those of base asphalt. Within 5 days of aging time, the four components content varied obviously, and as the aging time extended, their change rates slowed down.
(4) The asphalt composition had a direct influence on its low-temperature cracking performance. Under heat and light together, whether the base asphalt or modified asphalt, the change trends of its ductility and component content were similar. Therefore, the ductility of asphalt aged by heat and light together for 15 days was suggested for use as the evaluation index of the low-temperature cracking performance of asphalt.

## Figures and Tables

**Table 1:** Properties of styrene–butadiene–styrene (SBS) modifier.

Type	Elongation at Break (%)	Character	Permanent Set (%)	Tensile Strength (MPa)	Modulus at 300% Elongation (MPa)	Block Ratio
501s modifier	≥800	White floc	≤42	≥14.2	≥2.3	31/69
4402 modifier	≥700	White strip	≤40	≥14.0	≥2.0	30/70

**Table 2:** Properties of base asphalt.

Properties		Criteria	SK-70	ZH-70	Methods
Ductility at 10 °C (cm)		≥20	45	26.7	T0604-2011 [22]
Viscosity at 135 °C (Pa.s)		≤3.0	0.554	0.632	T0625-2011 [22]
Rutting factor (kPa)		≥1.0	1.91	1.20	AASHTOT315
Penetration degree at 25 °C (0.1 mm)		60~80	72	71	T0605-2011 [22]
Penetration index		−1.5 to +1.0	0.8	0.4	T0604-2011 [22]
Softening point (°C)		≥47	48.4	48.6	T0606-2011 [22]
After the thin film oven test (TFOT) (163 °C, 5 h)	Mass loss (%)	±0.8	−0.3	−0.14	T0609-2011 [22]
	Ductility at 10 °C (cm)	≥6s	16	26	T0604-2011 [22]
	Penetration degree ratio at 25 °C (%)	≥61	80.3	73.6	T0605-2011 [22]

**Table 3:** Properties of SBS-modified asphalt.

Properties		Criteria	Linear-Modified Asphalt		Branched-Modified Asphalt		Methods
			ZH-70	SK-70	ZH-70	SK-70	
Ductility at 10 °C (cm)		≥20	89	84	77	73	T0604-2011 [28]
viscosity at 135 °C (Pa.s)		≤3.0	1.625	1.400	1.853	1.769	T0625-2011 [22]
Rutting factor (kPa)		≥1.0	2.23	2.35	1.86	1.97	AASHTOT315
Penetration degree at 25 °C (0.1 mm)		30~60	42	49	48	51	T0605-2011 [28]
Penetration index		≥0	0.2	0.9	0.4	0.2	T0604-2011 [28]
Softening point (°C)		≥ 60	61.5	63.2	60.5	62	T0606-2011 [28]
After the thin film oven test (TFOT) (163 °C, 5 h)	Mass loss (%)	± 0.8	0.6	−0.3	−0.2	−0.2	T0609-2011 [28]
	Ductility at 10 °C (cm)	≥20	65	49	54	50	T0604-2011 [28]
	Penetration degree ratio at 25 °C (%)	≥65	85.7	83.7	81.3	73.4	T0605-2011 [28]

**Table 4:** ZH-70 base asphalt composition.

Aging Time	Saturates (%)	Aromatics (%)	Resins (%)	Asphaltenes (%)
0 day	13.35	49.28	28.32	9.05
5 days	13.70	45.32	28.35	12.63
10 days	13.65	44.91	28.64	12.80
15 days	13.54	44.50	28.91	13.05

**Table 5:** ZH-70 linear-modified asphalt composition.

Aging Time	Saturates (%)	Aromatics (%)	Resins (%)	Asphaltenes (%)
0 day	12.67	45.31	26.97	15.05
5 days	12.58	43.82	25.62	17.98
10 days	12.61	43.54	25.50	18.35
15 days	12.67	43.33	25.49	18.51
